# Shared and Distinct Topologically Structural Connectivity Patterns in Autism Spectrum Disorder and Attention-Deficit/Hyperactivity Disorder

**DOI:** 10.3389/fnins.2021.664363

**Published:** 2021-06-11

**Authors:** Lu Qian, Yun Li, Yao Wang, Yue Wang, Xin Cheng, Chunyan Li, Xiwen Cui, Gongkai Jiao, Xiaoyan Ke

**Affiliations:** ^1^Child Mental Health Research Center, Nanjing Brain Hospital Affiliated of Nanjing Medical University, Nanjing, China; ^2^Department of Psychiatry, Wuxi Mental Health Center, Nanjing Medical University, Wuxi, China

**Keywords:** Autism spectrum disorder, attention-deficit/hyperactivity disorder, diffusion tensor imaging, white matter structural networks, topological properties

## Abstract

**Background:**

Previous neuroimaging studies have described shared and distinct neurobiological mechanisms between autism spectrum disorders (ASDs) and attention-deficit/hyperactivity disorder (ADHD). However, little is known about the similarities and differences in topologically structural connectivity patterns between the two disorders.

**Methods:**

Diffusion tensor imaging (DTI) and deterministic tractography were used to construct the brain white matter (WM) structural networks of children and adolescents (age range, 6–16 years); 31 had ASD, 34 had ADHD, and 30 were age- and sex-matched typically developing (TD) individuals. Then, graph theoretical analysis was performed to investigate the alterations in the global and node-based properties of the WM structural networks in these groups. Next, measures of ASD traits [Social Responsiveness Scale (SRS)] and ADHD traits (Swanson, Nolan, and Pelham, version IV scale, SNAP-IV) were correlated with the alterations to determine the functional significance of such changes.

**Results:**

First, there were no significant differences in the global network properties among the three groups; moreover, compared with that of the TD group, nodal degree (*Ki*) of the right amygdala (AMYG.R) and right parahippocampal gyrus (PHG.R) were found in both the ASD and ADHD groups. Also, the ASD and ADHD groups shared four additional hubs, including the left middle temporal gyrus (MTG.L), left superior temporal gyrus (STG.L), left postcentral gyrus (PoCG.L), and right middle frontal gyrus (MFG.R) compared with the TD group. Moreover, the ASD and ADHD groups exhibited no significant differences regarding regional connectivity characteristics. Second, the ADHD group showed significantly increased nodal betweenness centrality (*Bi*) of the left hippocampus (HIP.L) compared with the ASD group; also, compared with the ADHD group, the ASD group lacked the left anterior cingulate gyrus (ACG.L) as a hub. Last, decreased nodal efficiency (*Enodal*) of the AMYG.R, *Ki* of the AMYG.R, and *Ki* of the PHG.R were associated with higher SRS scores in the ASD group. Decreased *Ki* of the PHG.R was associated with higher SRS scores in the full sample, whereas decreased *Bi* of the PHG.R was associated with lower oppositional defiance subscale scores of the SNAP-IV in the ADHD group, and decreased *Bi* of the HIP.L was associated with lower inattention subscale scores of the SNAP-IV in the full sample.

**Conclusion:**

From the perspective of the topological properties of brain WM structural networks, ADHD and ASD have both shared and distinct features. More interestingly, some shared and distinct topological properties of WM structures are related to the core symptoms of these disorders.

## Introduction

Autism spectrum disorder (ASD) and attention-deficit/hyperactivity disorder (ADHD) are both neurodevelopmental disorders that manifest early in life. ASD is defined by core symptoms of persistent and pervasive deficits in social communication and interaction along with repetitive behavioral patterns and restricted interests or activities. ADHD is characterized by developmentally inappropriate levels of inattention, impulsivity, and hyperactivity (American Psychiatric Association, 2013).

Aside from distinctive features, overlaps in the clinical symptoms and the genetic traits of ASD and ADHD are well documented. First, in terms of clinical phenotypes, 30 to 80% of all ASD children met the diagnostic criteria for ADHD, and 20 to 50% of children diagnosed with ADHD also met the diagnostic criteria for ASD (Van der Meer et al., 2012). Second, from a genetic point of view, genome-wide association studies and linkage or candidate gene studies also identified a number of genetic risk variants common to both disorders (Rommelse et al., 2010; Stergiakouli et al., 2017; Gudmundsson et al., 2019).

Brain phenotypes serve as a bridge to understand the clinical symptoms and biological mechanisms of disorders. Although previous studies have endeavored to verify whether there are similarities and differences in brain phenotypes between ASD and ADHD, the results have been inconsistent. A newly published study from 151 cohorts worldwide using structural T1-weighted whole-brain magnetic resonance imaging (MRI) data revealed ASD-specific cortical thickness differences in the frontal cortex of adult patients and ADHD-specific subcortical differences in children and adolescents; notably, the researchers did not find shared differences across the two disorders (Boedhoe et al., 2020). A diffusion tensor imaging (DTI) study applied tract-based spatial statistics to a larger sample (*n* = 200) of school-aged children with ASD and ADHD compared with typically developing (TD) controls and found that decreased fractional anisotropy (FA) within the splenium of the corpus callosum was common among the three groups (Ameis et al., 2016). Evidence from an functional MRI (fMRI) study (*n* = 1,305) measuring functional connectivity of the brain network also confirmed shared dysfunctional connectivity in the default mode network, dorsal attention network, and salience network of both ASD and ADHD patients between 7 and 21 years of age (Kernbach et al., 2018). Interestingly, another fMRI study of 56 children with ASD, 45 children with ADHD, and 50 TD children exhibited shared and distinct intrinsic functional network centrality between children with ASD and children with ADHD. Some affected areas were common to both groups, such as the precuneus; other affected areas were disorder-specific and included ADHD-related increases in degree centrality in the right striatum/pallidum, in contrast to ASD-related increases in bilateral temporolimbic areas specifically (Di Martino et al., 2013). Inconsistencies between previous findings might be due to participant heterogeneity, statistical power, or methods used.

Independent studies of ADHD and ASD have increasingly emphasized the role of dysconnectivity in large-scale networks in both disorders (Konrad and Eickhoff, 2010; Travers et al., 2012). As the brain is a complex network of structurally and functionally interconnected regions (Bullmore and Sporns, 2009), evaluating the brain as a whole and studying its networks can help us fully delineate the organizational patterns of internal connectivity in the human brain (Bullmore and Sporns, 2012).

Previous network studies have mainly focused on functional networks, while functional connectivity opens the door to the analysis of brain networks. We should not ignore that functional connections need structural support to make sense. DTI can help us map the structural connectivity between gray matter (GM) regions using white matter (WM) tractography, providing an avenue to probe structurally interconnected brain networks (Hagmann et al., 2008).

Accordingly, in the present study, we analyzed DTI data from 95 children and adolescents aged 6 to 16 years with ASD, with ADHD, and who were TD to map their WM structural networks. First, we investigated the alterations in the global and regional properties of the WM structural networks in these groups. Second, we examined the relationship between the alterations and measures of ASD traits, as well as ADHD traits, to determine the functional significance of such changes. To our knowledge, no previous studies have directly contrasted the global and regional properties of WM structural networks in individuals with ASD, individuals with ADHD, and TD controls. Our work might be an important supplement to previous studies.

## Materials and Methods

### Participants and Assessments

Patients were recruited via the Nanjing Brain Hospital affiliated with Nanjing Medical University, and controls were volunteers recruited through advertising on the hospital website and WeChat official account. In total, 95 participants aged 6 to 16 years were enrolled in our study, including 31 with ASD, 34 with ADHD, and 30 TD controls. Written informed consent was obtained from all legal guardians. All participants were age and sex matched. The intelligence quotient (IQ) scores of all participants were evaluated using the Wechsler Intelligence Scale for Children-IV (Feis, 2010), and those who scored less than 80 on the estimated full-scale IQ were excluded. The diagnoses for ASD and ADHD were based on the *Diagnostic and Statistical Manual of Mental Disorders, Fourth Text Revision* (*DSM-IV-TR*) diagnostic criteria supported by parent interviews, direct observations, available teacher forms, and prior records. ASD diagnosis was supported by standardized clinical assessments, including the Autism Diagnostic Observation Scale (Lord et al., 1989) and Autism Diagnostic Inventory – Revised (Lord et al., 1994). TD controls had no developmental disorders and no first-degree family history of such disorders. All participants with any systemic diseases, with a family history of head injury, with genetic syndromes, currently using antipsychotics, or with neurological disorders or psychiatric illness were excluded from the study. To discern brain–behavior relationships, we used parent ratings of ASD traits indexed by the Social Responsiveness Scale (SRS) (Constantino, 2013), which is a 65-item questionnaire covering each of the three *DSM-IV* criterion domains (social; language; and repetitive, stereotypic behaviors/restricted range of interest). ADHD traits were indexed by Swanson, Nolan, and Pelham-IV (SNAP-IV) (Gau et al., 2008), which is a widely used scale that measures the core symptoms of ADHD.

### Image Acquisition and Preprocessing

MRI data were acquired using the 3.0-T Verio MRI system (Siemens Medical Systems, Germany) with a birdcage gradient head coil. The head of each participant was gently restrained with foam cushions to avoid the generation of motion artifacts during the scan. High-resolution T1-weighted images were obtained using a three-dimensional spoiled gradient recalled pulse sequence with the following scanning parameters: repetition time (TR) = 2,530 ms, echo time (TE) = 3.34 ms, flip angle = 7°, inversion time = 1,100 ms, field of view (FOV) = 256 × 256 mm, matrix = 256 × 159, slice thickness = 1.33 mm, and total scanning time = 8.7 min. Each participant’s head was positioned parallel to the anterior commissure–posterior commissure plane. DTI was performed with single-shot echo planar imaging sequences with diffusion gradients applied in 30 non-collinear directions and *b* = 1,000 s/mm^2^. The thickness of each slice was 2.5 mm without a gap. The sequence parameters for the DTI were as follows: TE = 104 ms, TR = 9,000 ms, FOV = 230 × 230 mm^2^, and acquisition matrix = 128 × 128. The total DTI scanning time was 5.1 min. Before preprocessing, all the structural images were checked for artifacts. DWI image data were screened for subject motion and common artifacts related to diffusion sequences using PANDA (Cui et al., 2013), a pipeline toolbox for analyzing brain diffusion images. In brief, the overall preprocessing pipeline comprised the following steps: converting DICOM files into NIfTI images, estimating the brain mask (the b0 image without diffusion weighting was used for the estimation), cropping the raw images, correcting for the eddy-current effect, and averaging multiple acquisitions, which are detailed in PANDA (Cui et al., 2013).

### Network Construction

#### Network Node Definition

Node definition is an important step in brain network construction as the node is a vital element of a network (Sporns et al., 2005). Typically, the entire brain is divided into multiple regions using a prior GM atlas, where each region represents a network node (Bullmore and Sporns, 2009). In the present study, we used the automated anatomical labeling (AAL) atlas (Tzourio-Mazoyer et al., 2002) to parcellate the cerebral cortex into 90 regions (45 for each hemisphere, see Table 1 for details), and each region represents a node of the cortical network. As the parcellation process was conducted in the DTI native space for each subject, the individual FA image in native space was coregistered to its corresponding T1-weighted image using an affine transformation, and the individual resultant T1-weighted image was then non-linearly registered to the ICBM152 template in MNI space. Then, a prior atlas in standard space could be inversely warped back to the individual native space by applying the inverse warping transformation.

**TABLE 1:** The regions are listed in terms of a prior template of the automated anatomical labeling atlas.

**Index**	**Regions**	**Abbreviation**	**Index**	**Regions**	**Abbreviation**
(1,2)	Precental gyrus	PreCG	(47,48)	Lingual gyrus	LING
(3,4)	Superior frontal gyrus, dorsolateral	SFGdor	(49,50)	Superior occipital gyrus	SOG
(5,6)	Superior frontal gyrus, orbital part	ORBsup	(51,52)	Middle occipital gyrus	MOG
(7,8)	Middle frontal gyrus	MFG	(53,54)	Inferior occipital gyrus	IOG
(9,10)	Middle frontal gyrus, orbital part	ORBmid	(55,56)	Fusiform gyrus	FFG
(11,12)	Inferior frontal gyrus, opercular part	IFGoperc	(57,58)	Postcentral gyrus	PoCG
(13,14)	Inferior frontal gyrus, triangular part	IFGtriang	(59,60)	Superior parietal gyrus	SPG
(15,16)	Inferior frontal gyrus, orbital part	ORBinf	(61,62)	Inferior parietal, but supramarginal and angular gyri	IPL
(17,18)	Rolandic operculum	ROL	(63,64)	Supramarginal gyrus	SMG
(19,20)	Supplementary motor area	SMA	(65,66)	Angular gyrus	ANG
(21,22)	Olfactory cortex	OLF	(67,68)	Precuneus	PCUN
(23,24)	Superior frontal gyrus, media	SFGmed	(69,70)	Paracentral lobule	PCL
(25,26)	Superior frontal gyrus, medial orbital	ORBsupmed	(71,72)	Caudate nucleus	CAU
(27,28)	Gyrus rectus	REC	(73,74)	Lenticular nucleus, putamen	PUT
(29,30)	Insula	INS	(75,76)	Lenticular nucleus, pallidum	PAL
(31,32)	Anterior cingulate and paracingulate gyri	ACG	(77,78)	Thalamus	THA
(33,34)	Median cingulate and paracingulate gyri	DCG	(79,80)	Heschl gyrus	HES
(35,36)	Posterior cingulate gyrus	PCG	(81,82)	Superior temporal gyrus	STG
(37,38)	Hippocampus	HIP	(83,84)	Temporal pole: superior temporal gyrus	TPOsup
(39,40)	Parahippocampal gyrus	PHG	(85,86)	Middle temporal gyrus	MTG
(41,42)	Amygdala	AMYG	(87,88)	Temporal pole: middle temporal gyrus	TPOmid
(43,44)	Calcarine fissure and surrounding cortex	CAL	(89,90)	Inferior temporal gyrus	ITG

*The regions are listed in terms of a prior template of an automated anatomical labeling atlas (Tzourio-Mazoyer et al., 2002).*

#### Network Edge Definition

Deterministic tractography was used to define the network edges of the 90 regions. FA thresholding was set as 0.1–1, with an interval of 0.1, and the turning angle threshold was set at 35. In this study, we selected a threshold value of 3 (*T* = 3) for the number of fiber bundles, meaning two regions were considered structurally connected if at least three fibers with two endpoints were located in these two regions. Such a threshold selection was reported to reduce the risk of false-positive connections due to noise or limitations in deterministic tractography (Shu et al., 2011). After defining the network edges, PANDA was used to calculate the fiber number (FN)–weighted WM network for each participant, which was represented by a symmetric 90 × 90 matrix.

#### Network Analysis

We investigated the topological properties of the WM structural networks at the global and nodal levels. Characteristic path length (*Lp*), clustering coefficient (*Cp*), normalized shortest path length (λ), normalized clustering coefficient (γ), small-worldness σ (σ = λ/γ), local efficiency (*Eloc*), and global efficiency (*Eglob*) of the whole brain network were calculated to quantify the global network architecture. Also, we computed the local efficiency of node *i* (*Enodal*), nodal degree of node *i* (*Ki*), and betweenness centrality of node *i* (*Bi*) to determine the nodal (regional) characteristics of the networks. In addition, the normalized betweenness (*bi*) was used to identify the most central nodes (hubs) of the networks. These measures are detailed in the article by Rubinov and Sporns (2010). Briefly, *Lp* is the average shortest path length between the nodes that quantifies the ability of a network to propagate parallel information, and *Cp* represents the fraction of the node’s neighbors that are also neighbors of each other, λ = *Lp*^real^*/Lp*^rand^, γ = *Cp*^real^*/Cp*^rand^, where *Lp*^rand^ and *Cp*^rand^ are the mean shortest path length and the mean clustering coefficient of 100 matched random networks, respectively. *Eloc* is the mean of the local efficiency of all the nodes in the graph. *Eglob* is the average inverse shortest path length and can be used to estimate the efficiency with which brain regions communicate. *Enodal* represents the regional efficiency of a node. The degree *Ki* is defined as the number of connections to that node, and highly connected nodes have a large degree. *Bi* is defined as the fraction of all the shortest paths in the network that pass through a given node.

### Statistical Analysis

The statistics of demographic and clinical characteristics were conducted using Statistical Product and Service Solutions (SPSS, version 22.0). Normality of the distributions was assessed by the Shapiro–Wilk test. Categorical variables (sex) were investigated with χ^2^ tests, whereas continuous variables, such as age, IQ, clinical scale scores, and network properties, were investigated with one-way analysis of variance (ANOVA), followed by Bonferroni *post hoc* analysis. Effect size η*_p_*^2^ is reported for one-way ANOVA tests, with 0.01, 0.06, and 0.14 representing small, medium, and large effects, respectively.

To identify the hub regions of the networks between groups, we first calculated the *bi* of all 90 regions for each subject and then calculated the mean value of the *bi* for each region according to the groups. If *bi* was greater than 1.5 times the average betweenness (the mean value of *bi* for all 90 regions) of the network, the nodes were considered to be pivotal nodes (i.e., hubs).

Network-based statistics (NBS) (Zalesky et al., 2010) is a non-parametric method based on the principles of traditional cluster-based thresholding of statistical parametric maps to control the familywise error rate; hence, it can be used to identify subnetworks of topologically connected suprathreshold connections. We conducted an independent *F* test on every connectivity value to compare the structural connectivity differences between the ASD, ADHD, and TD groups. We set the primary threshold at *p* = 0.001 for the resulting *F* statistic matrix (based on a height threshold of *F* = 3.2 to stringently control for type I errors). Next, the data across groups were randomized 5,000 times to obtain the reference cluster distribution. We used the maximum number of connections across all clusters to form the reference distribution for each randomization; cluster scores exceeding the 95th percentile were considered significant (*p* < 0.05). For *post hoc* paired comparisons (e.g., the ASD group vs. the ADHD group), the procedure was similar.

Additionally, Pearson correlation analysis, performed in SPSS, was used to examine correlations in the full sample, and *post hoc* analyses examined Pearson correlations in the patient groups to reveal the relationships between altered regional properties and ASD traits, as well as between altered regional properties and ADHD traits.

## Results

### Demographic and Clinical Characteristics

One-way ANOVAs indicated that the three groups were matched for age and sex but not matched for IQ (*F* = 4.979, *p* = 0.009, η*_p_*^2^ = 0.098) (it should be noted that the IQ effect was removed in all of the following network analyses). The ANOVAs showed significant group effects on SRS total scores (*F* = 13.236, *p* = 0.000, η*_p_*^2^ = 0.223) and SNAP-IV subscale scores, including inattention (*F* = 65.038, *p* = 0.000, η*_p_*^2^ = 0.591), hyperactivity (*F* = 40.230, *p* = 0.000, η*_p_*^2^ = 0.472), and oppositional defiance (*F* = 10.626, *p* = 0.000, η*_p_*^2^ = 0.191). Specifically, for pairwise comparisons, the ASD group showed greater social deficits than the ADHD and TD groups; moreover, the ADHD group showed more severe ADHD symptoms than the ASD and TD groups (see Table 2 for details).

**TABLE 2:** The demographic and clinical characteristics of all participants.

	**ASD (*n* = 31)**	**ADHD (*n* = 34)**	**TD (*n* = 30)**	***p* value^*a*^**	**Pairwise^*b*^**
Gender: male/female	27/4	32/2	22/8	0.051	–
Age (std)	9.00 (1.92)	9.44 (1.67)	9.67 (2.88)	0.483	–
IQ (std)	102.26 (19.70)	104.09 (15.56)	107.07 (18.43)	0.009*	ASD vs. ADHD, n.s. ASD vs. TD, *p* = 0.014 ADHD vs. TD, *p* = 0.036
**SRS-total^*c*^ (std)**	64.65 (15.77)	51.71 (13.60)	46.43 (8.54)	0.000*	ASD vs. ADHD, *p* = 0.000* ASD vs. TD, *p* = 0.000* ADHD vs. TD, n.s.
**SNAP-IV^*d*^ subscales**					
Inattention (std)	9.35 (2.24)	17.06 (3.82)	7.24 (4.44)	0.000*	ASD vs. ADHD, *p* = 0.000* ASD vs. TD, n.s. ADHD vs. TD, *p* = 0.000*
Hyperactivity (std)	6.97 (2.52)	12.58 (5.21)	3.79 (3.36)	0.000*	ASD vs. ADHD, *p* = 0.000* ASD vs. TD, *p* = 0.007* ADHD vs. TD, *p* = 0.000*
Oppositional defiant (std)	4.13 (2.99)	8.55 (5.22)	5.31 (3.18)	0.000*	ASD vs. ADHD, *p* = 0.000* ASD vs. TD, n.s. ADHD vs. TD, *p* = 0.006*

*p < 0.05. std: standard deviation; n.s.: no significance.

*^*a*^*p* value for sex using the χ^2^test; other *p* values for the comparisons based on one-way ANOVA.*

*^*b*^*Post hoc* pairwise comparisons were then performed using the *t* test.*

*^*c*^Total scores based on the Social Responsiveness Scale.*

*^*d*^Swanson, Nolan, and Pelham, version IV scale.*

### Global Topological Properties of the WM Structural Networks

Based on the constructed networks, we calculated the topological properties (i.e., *Lp*, *Cp*, λ, γ, σ, *Eloc*, and *Eglob*) of the global network for each participant and showed the mean values of these properties of the three groups. Analyses of covariance (ANCOVAs) on the global network properties showed no significant group effects of all the global topological properties (*Lp*: *F* = 1.625, *p* = 0.202, η*_p_*^2^ = 0.034; *Cp*: *F* = 0.666, *p* = 0.516, η*_p_*^2^ = 0.014; λ: *F* = 0.583, *p* = 0.560, η*_p_*^2^ = 0.013; γ: *F* = 1.253, *p* = 0.290, η*_p_*^2^ = 0.027; σ: *F* = 2.343, *p* = 0.078, η*_p_*^2^ = 0.072; *Eloc*: *F* = 1.195, *p* = 0.308, η*_p_*^2^ = 0.025; and *Eglob*: *F* = 2.535, *p* = 0.085, η*_p_*^2^ = 0.052) among the three groups.

### Node-Based Analysis of the WM Structural Networks

#### Identification of Regional Property Differences

**FIGURE 1:**
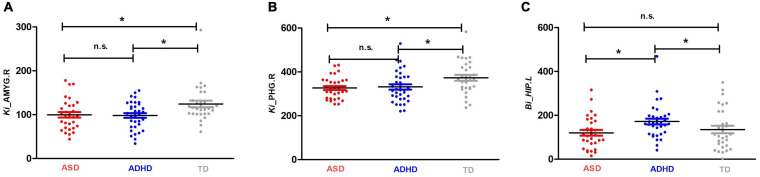
Shared and specific nodal topological properties in diagnostic groups. **(A,B)** represent the regions with shared deficits in ASD and ADHD topological properties compared with the properties of TD controls, and **(C)** represents the region in which ADHD topological properties differ from both TD controls and individuals with ASD by Bonferroni *post hoc* analysis; for the definitions of the abbreviations, see Table 1. For each node, the bar and error bar represent the mean value and SD, respectively; a single asterisk (*) represents a significant group difference at *p* < 0.05; n.s. represents no significance.

*Enodal*, *Ki*, and *Bi* were calculated to identify the differences in nodal properties in the participants. Among the three groups, ANCOVAs showed that the regions with significant group effects were distributed in the temporal [the left amygdala (AMYG.L), right amygdala (AMYG.R), left hippocampus (HIP.L), and right parahippocampal gyrus (PHG.R)], and subcortical [the right insula (INS.R)] cortices (see Table 3 for details). *Post hoc* tests showed that the *Ki* values of the AMYG.R and PHG.R were significantly decreased in both the ASD and ADHD groups compared with the TD controls (i.e., ASD and ADHD shared deficits). Only one region (i.e., the HIP.L) showed significant group differences, with a higher *Bi* observed in the ADHD group than in the ASD and TD groups (i.e., ADHD-specific deficits) (Figure 1).

**TABLE 3:** Regions showing significant differences in the nodal topological properties among the ASD, ADHD, and TD groups.

**Nodal metrics**	**ASD**		**ADHD**		**TD**		***F***	***p* value^*a*^**	**η*_p_*^2^*^*b*^***
	**Mean**	**std**	**Mean**	**std**	**Mean**	**std**			
*Enodal_*AMYG.R	10.31	1.98	10.44	2.01	11.73	2.44	3.266	0.025*	0.097
*Ki_* PHG.R	326.5	50.39	331.6	71.76	373.2	75.50	3.684	0.015*	0.108
*Ki_* AMYG.L	105.7	31.90	103.7	27.19	124.4	35.62	2.946	0.037*	0.089
*Ki_* AMYG.R	99.71	34.90	98.26	31.62	124.3	41.42	5.278	0.002*	0.148
*Bi_*INS.R	611.20	206.30	528.80	228.40	482.50	157.90	2.914	0.039*	0.088
*Bi_*HIP.L	120.20	73.08	171.70	78.97	135.40	94.14	2.714	0.049*	0.082
*Bi_*PHG.R	134.60	59.08	143.50	98.06	196.30	113.20	2.890	0.040*	0.087

*p < 0.05. std: standard deviation; for the definitions of the abbreviations, see Table 1.

*^*a*^*p* value for using one-way ANCOVA tests.*

*^*b*^η*_p_*^2^: effect size for one-way ANCOVA tests.*

#### Identification of Hub Distributions

**FIGURE 2:**
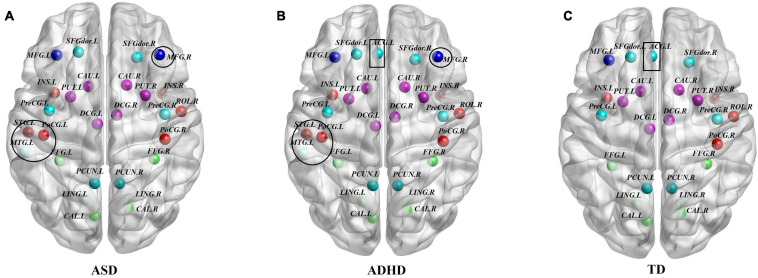
Comparisons of hub distributions among the ASD, ADHD, and TD groups. **(A)** ASD hub distributions, **(B)** ADHD hub distributions, **(C)** TD hub distributions; for the definitions of the abbreviations, see Table 1; regions in the black circle including the MTG.L, STG.L, PoCG.L, and MFG.R were additional hubs of both the ASD and ADHD groups compared with the TD controls. In addition, the ACG.L in the black rectangle was identified as a hub in the ADHD and TD groups but not in the ASD group. The regions were mapped onto the cortical surfaces using BrainNet viewer software (www.nitrc.org/projects/bnv/). Note that the FN-weighted WM network for each participant was constructed using the AAL template.

To identify the hub regions, we examined the *Bi* of each node in all networks. In total, 23 hub regions were the same for all of the groups, including 10 regions of the association cortex [the left precuneus (PCUN.L), right precuneus (PCUN.R), left fusiform gyrus (FFG.L), right fusiform gyrus (FFG.R), left lingual gyrus (LING.L), right lingual gyrus (LING.R), left dorsolateral superior frontal gyrus (SFGdor.L), right dorsolateral superior frontal gyrus (SFGdor.R), left middle frontal gyrus (MFG.L), and right rolandic operculum (ROL.R)], four regions of subcortical structures [the left putamen (PUT.L), right putamen (PUT.R), left caudate nucleus (CAU.L), and right caudate nucleus (CAU.R)], four paralimbic regions [the left median cingulate gyrus (DCG.L), right median cingulate gyrus (DCG.R), left insula (INS.L), and INS.R], and five primary regions [the left calcarine fissure (CAL.L), right calcarine fissure (CAL.R), left precental gyrus (PreCG.L), right precental gyrus (PreCG.R), and right postcentral gyrus (PoCG.R)]. The common hubs identified for all of the groups were predominantly distributed in the association cortices. Specially, the left anterior cingulate gyrus (ACG.L) was identified as a hub in the ADHD and TD groups but not in the ASD group, and both the ASD and ADHD groups exhibited four additional hubs [the left middle temporal gyrus (MTG.L), left superior temporal gyrus (STG.L), left postcentral gyrus (PoCG.L), and right middle frontal gyrus (MFG.R)] compared with TD controls (Table 4 and Figure 2).

**TABLE 4:** Hub distributions of the WM structural networks among the ASD, ADHD, and TD groups.

**ASD**		**ADHD**		**TD**	
**Regions**	**Class^*a*^**	**Regions**	**Class^*a*^**	**Regions**	**Class^*a*^**
PCUN.R	Association	PCUN.R	Association	PCUN.R	Association
DCG.L	Paralimbic	PCUN.L	Association	PCUN.L	Association
PCUN.L	Association	DCG.L	Paralimbic	DCG.L	Paralimbic
DCG.R	Paralimbic	INS.R	Paralimbic	DCG.R	Paralimbic
INS.R	Paralimbic	DCG.R	Paralimbic	CAL.R	Primary
INS.L	Paralimbic	INS.L	Paralimbic	INS.L	Paralimbic
PoCG.R	Primary	PoCG.R	Primary	INS.R	Paralimbic
PreCG.R	Primary	CAL.R	Primary	FFG.L	Association
CAL.R	Paralimbic	CAL.L	Primary	CAL.L	Primary
FFG.L	Association	CAU.L	Subcortical	FFG.R	Association
PUT.R	Paralimbic	ROL.R	Association	PUT.R	Subcortical
FFG.R	Association	MFG.L	Association	LING.L	Association
CAL.L	Primary	PUT.R	Subcortical	PreCG.L	Primary
CAU.R	Primary	PreCG.L	Primary	MFG.L	Association
MFG.L	Association	SFGdor.L	Association	PoCG.R	Primary
ROL.R	Association	FFG.L	Association	SFGdor.L	Association
SFGdor.L	Association	CAU.R	Subcortical	LING.R	Association
CAU.L	Paralimbic	FFG.R	Association	PreCG.R	Primary
LING.R	Association	PUT.L	Subcortical	CAU.L	Subcortical
PUT.L	Paralimbic	SFGdor.R	Association	PUT.L	Subcortical
SFGdor.R	Association	MFG.R	Association	SFGdor.R	Association
MTG.L	Association	LING.L	Association	CAU.R	Subcortical
PreCG.L	Primary	ACG.L	Paralimbic	ROL.R	Association
STG.L	Association	LING.R	Association	ACG.L	Paralimbic
MFG.R	Association	PreCG.R	Primary		
LING.L	Association	MTG.L	Association		
PoCG.L	Primary	STG.L	Association		
		PoCG.L	Primary		

*For the definitions of the abbreviations, see Table 1.*

*^*a*^The cortical regions are classified as primary, association, subcortical, limbic, and paralimbic (Supekar et al., 2009).*

### Connectivity-Based Analysis

**FIGURE 3:**
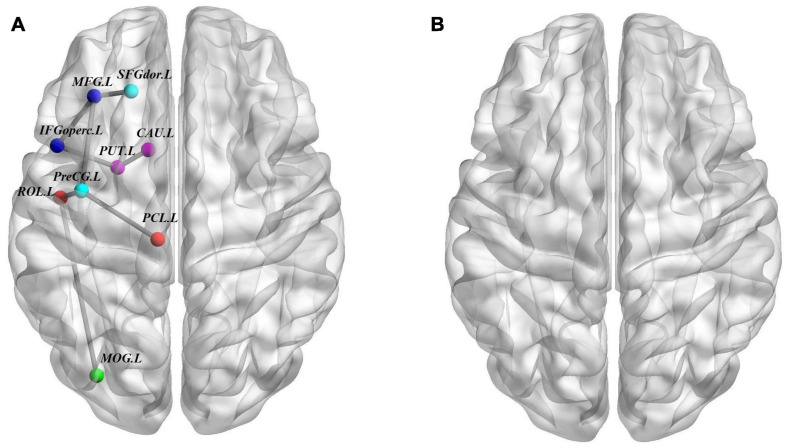
Structural connectivity (SC) differences. **(A)** The structural connectome network differences among ASD, ADHD, and TD groups identified by NBS; these connections formed a single connected network with nine nodes and eight connections (*p* = 0.001, corrected). **(B)** Similar to A but between ASD and ADHD groups, no connected components with significant differences were found. For the definitions of the abbreviations, see Table 1. The nodes and connections were mapped onto the cortical surfaces using BrainNet viewer software (www.nitrc.org/projects/bnv/). Note that the FN-weighted WM network for each participant was constructed using the AAL template.

We used the NBS method to identify the disrupted connected components among the three groups, and we found that a connected network with nine nodes and eight edges was altered (*p* = 0.001, corrected). The involved nodal regions were the PreCG.L, left middle frontal gyrus (MFG.L), SFGdor.L, left inferior frontal gyrus, opercular part (IFGoperc.L), left rolandic operculum (ROL.L), left middle occipital gyrus (MOG.L), left paracentral lobule (PCL.L), PUT.L, and CAU.L. However, no significant differences were found with respect to connected components between the ASD and ADHD groups (Figure 3).

### Correlation of Altered Regional Properties With ASD and ADHD Traits

**FIGURE 4:**
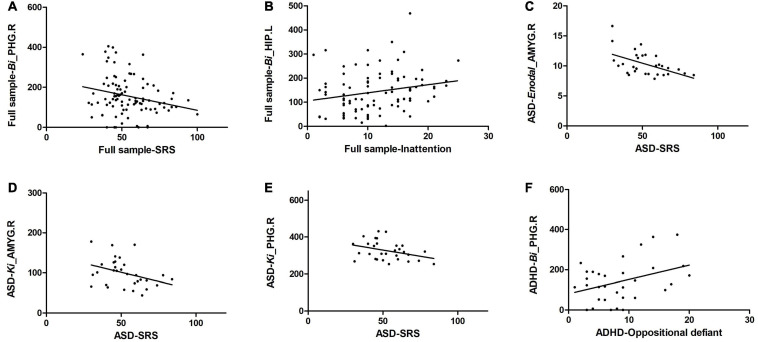
Significant correlation of altered regional properties with ASD and ADHD traits. **(A)** *Bi* of the PHG.R was significantly correlated with SRS scores in the full sample; **(B)** *Bi* of the HIP.L was significantly correlated with inattention subscale scores of the SNAP-IV in the full sample; **(C)** *Enodal* of the AMYG.R was significantly correlated with SRS scores in the ASD group; **(D)** *Ki* of the AMYG.R was significantly correlated with SRS scores in the ASD group; **(E)** *Ki* of the PHG.R was significantly correlated with SRS scores in the ASD group, and **(F)** *Bi* of the PHG.R was significantly correlated with oppositional defiance subscale scores of the SNAP-IV in the ADHD group.

Correlation analysis between altered regional properties and ASD traits and that between altered regional properties and ADHD traits were examined in the full sample and in the patient groups. For regional properties, we examined the nodes with significant group effects that are listed in Table 3. In the full sample, *Bi* of the PHG.R was significantly correlated with SRS scores (*r* = −0.242, *p* = 0.018, *r*^2^ = 0.059), and *Bi* of the HIP.L was significantly correlated with inattention subscale scores of the SNAP-IV (*r* = 0.220, *p* = 0.034, *r*^2^ = 0.048). In the ASD group, *Enodal* of the AMYG.R was significantly correlated with SRS scores (*r* = −0.512, *p* = 0.003, *r*^2^ = 0.262; also, *Ki* of the AMYG.R was significantly correlated with SRS scores (*r* = −0.359, *p* = 0.005, *r*^2^ = 0.128), and *Ki* of the PHG.R was significantly correlated with SRS scores (*r* = −0.368, *p* = 0.004, *r*^2^ = 0.135). In the ADHD group, *Bi* of the PHG.R was significantly correlated with oppositional defiance subscale scores of the SNAP-IV (*r* = 0.374, *p* = 0.032, *r*^2^ = 0.140) (Figure 4).

## Discussion

The present study compared the global and regional properties of the WM structural networks in children and adolescents with ASD, ADHD, and TD controls; the findings suggest shared and distinct features underlying neurobiological mechanisms in ASD and ADHD children and adolescents from a network perspective.

The human brain is a complex system with an optimal balance between local specialization and global integration (Shu et al., 2011). In this study, we identified the global topological properties of the WM networks in ASD and ADHD patients and TD controls, exhibiting no significant differences among the three groups. Our results are supported by a network study that showed measures of structural *Cp* and *Lp* did not significantly differ between children and adolescents with ASD and TD controls (Rudie et al., 2013). However, a recent work exploring the topologic architecture of WM connectivity networks in preschool-aged children demonstrated that children with ASD had shortened *Lp* and increased *Eglob* and *Cp* compared with the TD children (Li et al., 2018). Another study focused on wiring of the connectome in adults with high-functioning ASD showed that *Eglob* was significantly decreased, and *Lp* was significantly increased in subjects with ASD (Roine et al., 2015). In addition, consistent with our results, Justina et al., in an investigation of the organization of structural brain networks in adults with ADHD and unaffected controls, also showed no significant differences in global network metrics, including σ, *Eglob*, and *Cp* (Sidlauskaite et al., 2015). However, Chen et al. (2019) demonstrated altered topological characteristics of lower *Eglob*, lower *Eloc*, and longer *Lp* of brain functional networks in drug-naive children with ADHD, whereas in the other two studies using drug-treated samples, no significant changes in *Eglob* in ADHD subjects were found (Wang et al., 2009; Xia et al., 2014). Therefore, age range and medication effects might contribute to the differences in findings regarding the global topological properties of brain networks in patients with ASD and those with ADHD.

Shared deficits of regional alterations were associated with a tendency of decreased *Ki* of the AMYG.R and PHG.R in the ASD and ADHD groups. Converging neuroscientific evidence has suggested that the neuropathology of ASD is widely distributed, involving impaired connectivity throughout the brain (Just et al., 2004; Ameis and Catani, 2015). One region consistently highlighted by structural MRI (Gibbard et al., 2017) and fMRI (Guo et al., 2016; Odriozola et al., 2018; Iidaka et al., 2019) studies is the amygdala. Abnormal amygdala structure and function are correlated with alterations in the social–emotional functions of ASD in rodents (Schoch et al., 2016) and in humans (Gotts et al., 2012; Mundy, 2017). The commonality of amygdala abnormalities has also been identified in ADHD (Posner et al., 2011; Hulvershorn et al., 2014), and altered amygdala activation and connectivity have been suggested to be related to dysfunction of emotion regulation in ADHD (Christiansen et al., 2019, such as facial and contextual emotion processing (Fonseca et al., 2009; Miller et al., 2011). A recent structural neuroimaging study directly comparing amygdala volumes and correlates of social deficits showed that larger amygdala volumes were associated with fewer social deficits in both ASD and ADHD children, supporting a common underlying biology between these disorders (Baribeau et al., 2019). Additionally, the PHG is thought to be a structure of the default mode network (Fox et al., 2005) known to be particularly active when participants are at rest, and it has been identified as a key anatomical region in the mesial temporal lobe associated with memory (Aggleton et al., 2010; Catani et al., 2013). One study reported that the PHG was involved in the involuntary reactivation of contextual fear memory that results in avoidance (Paquette et al., 2003). Moreover, it is a part of the limbic system, which was mentioned as a socially related area of the brain (Pagani et al., 2012; Catani et al., 2013). Several ASD (Monk et al., 2009; Weng et al., 2010; Lee et al., 2016) and ADHD (Anderson et al., 2014) studies have revealed altered functional connectivity between the PHG and other brain regions compared with the functional connectivity of TD controls. Our work suggests shared deficits of *Ki* in the PHG.R in the ASD and ADHD groups.

A novel finding in this study was the identification of an increased tendency of *Bi* of the HIP.L in the ADHD group compared with the ASD and TD groups. Studies from animal models of ADHD have suggested abnormalities in neuronal signaling systems within the hippocampus (Medin et al., 2013; Sterley et al., 2013). A multimodal MRI study in children with ADHD showed that hippocampal volumes were reduced in children with ADHD and that hippocampal–orbitofrontal cortex connectivity was also reduced in children with ADHD (Posner et al., 2014). Another study also confirmed reduced hippocampal volume in children with ADHD (Hoogman et al., 2017). Furthermore, a study showed that synaptic plasticity of neurons in the hippocampus may contribute to learning and memory processes (Filippo et al., 2009), and dysfunction of the hippocampus could affect learning processes and further result in ADHD symptoms. However, in terms of ASD, Groen et al. (2010) showed that the HIP.L was significantly enlarged in adolescents with autism compared with that of the control group. Schumann et al. (2004) also revealed enlarged hippocampi, especially in a high-functioning autism group of 7.5- to 12.5-year-olds. In agreement with previous studies showing structural differences of the hippocampus between ADHD and ASD patients, our findings suggest that regional characteristics of the hippocampus might be a valuable brain network marker for distinguishing ADHD from ASD.

In terms of hubs, we used the index of *Bi* to identify the hub regions of the WM networks in the present study. Surprisingly, the distributions of hubs in the ASD and ADHD groups were almost the same. We found that most of the hubs were located in the association cortex, which plays a central role in receiving convergent inputs from multiple cortical regions (Mesulam, 1998). These findings are in accordance with several previous studies of ASD (Takashi et al., 2014; Qian et al., 2018) and ADHD (Sidlauskaite et al., 2015), which identified the association cortex a critical node in both structural and functional brain networks. Moreover, compared with the TD control group, both the ASD and ADHD groups exhibited common alterations in hub distributions with four additional regions, including the MTG.L, STG.L, PoCG.L, and MFG.R. ASD subjects were found to have similar hub alterations comparable with ADHD subjects, particularly in the frontal and temporal regions, which is in accordance with the results of previous meta-analyses and mega-analyses (Van Rooij et al., 2018; Hoogman et al., 2019). According to the hub results, we found that the ASD group lacked one hub region of the ACG.L compared with the ADHD and TD groups. As hub regions are believed to handle multimodal or integrative functions, damage to these regions could dramatically affect the stability and efficiency of the network (Achard et al., 2006). A 4-year longitudinal study in healthy control participants reported that greater age-related thinning of the left anterior cingulate cortex was associated with less reduction in effortful control and in turn is associated with improvements in socioemotional functioning (Vijayakumar et al., 2014). Moreover, many studies performed in both humans and rodents have shown that the ACG is anatomically and functionally connected to a broad set of regions engaged in social information processing (Chang et al., 2012; Apps et al., 2016) and therefore is likely to represent an information hub for the social network (Guo et al., 2019). In addition, the ACG is part of the default mode network and is a primary node of the salience network; both networks are strongly implicated in autism (Zielinski et al., 2012). Although the exact etiopathogenesis of ASD remains unclear, a consistent biomarker could estimate patient impairment and guide tailored rehabilitation. Our study reveals that the hubness of the ACG might be a potential biomarker for the diagnosis of ASD, distinguishing it from ADHD and neurotypical development. Moreover, despite shared regional characteristics, we found common structural connectivity patterns of both the ASD and ADHD groups by NBS. We assumed that this might be partly related to the common distribution of hubs between the two groups. Evidence from Kernbach et al. (2018) also showed shared dysfunctional connectivity in the default mode network, dorsal attention network, and salience network in ASD and ADHD subjects, which is consistent with our work.

Numerous prior studies have addressed the relationship between the amygdala and autistic symptoms in ASD patients (Gotts et al., 2012; Mundy, 2017). Our correlation results were in accordance with previous studies revealing decreased *Ki* and *Enodal* of the AMYG.R that was associated with severe autistic symptoms in the ASD group. However, our correlation results failed to exhibit a significant association between the regional properties of the AMYG.R and SRS scores in the ADHD group. This outcome might be because ADHD subjects in our study showed no deficits that are considered ASD traits measured by the SRS. Moreover, our correlation results showed a decreased *Bi* of the PHG.R and *Ki* of the PHG.R; these values were also significantly correlated with severe autistic symptoms in the full sample and the ASD group, respectively. This common tendency is consistent with the findings of a previous study, which showed that the greater the degree of social impairment, the weaker the connectivity between the PHG and other brain regions (Weng et al., 2010). Notably, decreased *Bi* of the PHG.R was associated with better performance on the oppositional defiance subscale in the ADHD group, which might be an important complement to previous studies. Notably, our results revealed that abnormal *Bi* of the HIP.L was an ADHD-specific region in the brain network, and our correlation analysis further showed the relationship between altered *Bi* of the HIP.L and ADHD core symptoms (i.e., inattention) in the full sample; these findings suggest the unique role of the HIP.L in the pathogenesis of ADHD.

In conclusion, our findings demonstrate that *Bi* of the HIP.L and hubness of the ACG.L might be valuable markers for distinguishing ASD from ADHD. While disorder-specific abnormalities are present, overlapping results are consistent with the growing clinical, molecular, and neuroimaging evidence of commonalities (Rommelse et al., 2011). In the present study, we found shared *Ki* alterations of the AMYG.R and PHG.R, as well as shared global network properties, hub distributions, and regional connectivity in the ASD and ADHD groups. In addition, altered *Enodal* of the AMYG.R, *Ki* of the AMYG.R, *Bi* of the PHG.R, and *Ki* of the PHG.R were associated with autistic symptoms, and altered *Bi* of the PHG.R and *Bi* of the HIP.L were associated with ADHD symptoms. Several aspects of our study are intriguing. First, no previous studies have concentrated on comparing topological properties of WM structural networks in individuals with ASD, individuals with ADHD, and TD controls concurrently. Our study is an important complement to previous studies. Second, our study analyzed different domains of topological properties, including global-based and node-based (e.g., regional properties, hub distributions, and regional connectivity) properties, providing comprehensive evidence for both shared and distinct underlying mechanisms in ASD and ADHD children and adolescents. Third, identifying and validating the similarities and differences of brain-based endophenotypes in the two disorders may be beneficial for personalized medicine (Collins and Varmus, 2015).

## Limitations

There are several limitations that should be addressed. First, as mentioned previously, ASD and ADHD are neurodevelopmental disorders with overlapping clinical presentations (Van der Meer et al., 2012), while individuals without comorbid ADHD symptoms were included as ASD subjects in our study. Future analyses are required to explore the extent to which ADHD-like comorbidities in ASD share common neural correlates with ADHD. Second, this study used a deterministic tractography tracking procedure that produces more image noise and is of low resolution, making fiber tracking more error-prone because of the “fiber crossing” problem (Mori and van Zijl, 2002). The use of probabilistic tractography to define network edges could be helpful in addressing this issue (Iturria-Medina et al., 2008). Third, although the three groups did not differ significantly in sex distribution, most participants were male, reflecting the higher prevalence of boys with ASD and ADHD. Thus, our results may not generalize to girls, and future studies can enroll more female participants to further reveal sex differences in WM topological properties. Finally, the present participants were not matched for IQ; although the IQ effect was removed in all of the network analyses, these data should be interpreted with caution.

## Data Availability Statement

The raw data supporting the conclusions of this article will be made available by the authors, without undue reservation.

## Ethics Statement

The studies involving human participants were reviewed and approved by the Medical Ethics Committees of the Nanjing Brain Hospital Affiliated to Nanjing Medical University. Written informed consent to participate in this study was provided by the participants’ legal guardian/next of kin.

## Author Contributions

XK designed the study and revised the draft. LQ, YL, YaW, YuW, XC, CL, XC, and GJ collected and analyzed the data. LQ wrote the first draft of the manuscript. All authors contributed to and approved the final manuscript.

## Conflict of Interest

The authors declare that the research was conducted in the absence of any commercial or financial relationships that could be construed as a potential conflict of interest.

## References

[B1] Achard S. Salvador R. Whitcher B. Suckling J. Bullmore E. D. (2006). A resilient, low-frequency, small-world human brain functional network with highly connected association cortical hubs. *J. Neurosci.* 26 63–72. 10.1523/JNEUROSCI.3874-05.2006 16399673 PMC6674299

[B2] Aggleton J. P. O’Mara S. M. Vann S. D. Wright N. F. Tsanov M. Erichsen J. T. (2010). Hippocampal-anterior thalamic pathways for memory: uncovering a network of direct and indirect actions. *Eur. J. Neurosci.* 31 2292–2307. 10.1111/j.1460-9568.2010.07251.x 20550571 PMC2936113

[B3] Ameis S. H. Catani M. (2015). Altered white matter connectivity as a neural substrate for social impairment in autism spectrum disorder. *Cortex* 62 158–181. 10.1016/j.cortex.2014.10.014 25433958

[B4] Ameis S. H. Lerch J. P. Taylor M. J. Lee W. Viviano J. D. Pipitone J. (2016). A diffusion tensor imaging study in children with ADHD, autism spectrum disorder, OCD, and matched controls distinct and non-distinct white matter disruption and dimensional brain-behavior relationships. *Am. J. Psychiatry* 173 1213–1222. 10.1176/appi.ajp.2016.15111435 27363509

[B5] American Psychiatric Association (2013). *Diagnostic and Statistical Manual of Mental Disorders*, 5th Edn, Arlington, VA: American Psychiatric Association.

[B6] Anderson A. Douglas P. K. Kerr W. T. Haynes V. S. Yuille A. L. Xie J. (2014). Non-negative matrix factorization of multimodal MRI, fMRI and phenotypic data reveals differential changes in default mode subnetworks in ADHD. *Neuroimage* 102 207–219. 10.1016/j.neuroimage.2013.12.015 24361664 PMC4063903

[B7] Apps M. A. Rushworth M. F. Chang S. W. (2016). The anterior cingulate gyrus and social cognition: tracking the motivation of others. *Neuron* 90 692–707. 10.1016/j.neuron.2016.04.018 27196973 PMC4885021

[B8] Baribeau D. A. Dupuis A. Paton T. A. Hammill C. Scherer S. W. Schachar R. J. (2019). Structural neuroimaging correlates of social deficits are similar in autism spectrum disorder and attention-deficit/hyperactivity disorder: analysis from the pond network. *Transl. Psychiat.* 9:72. 10.1038/s41398-019-0382-0 30718456 PMC6361977

[B9] Boedhoe P. S. W. van Rooij D. Hoogman M. Twisk J. W. R. Schmaal L. Abe Y. (2020). Subcortical brain volume, regional cortical thickness, and cortical surface area across disorders: findings from the ENIGMA ADHD, ASD, and OCD working groups. *Am. J. Psychiatry* 177 834–843. 10.1176/appi.ajp.2020.19030331 32539527 PMC8296070

[B10] Bullmore E. Sporns O. (2009). Complex brain networks: graph theoretical analysis of structural and functional systems. *Nat. Rev. Neurosci.* 10:312. 10.1038/nrn2618 19190637

[B11] Bullmore E. Sporns O. (2012). The economy of brain network organization. *Nat. Rev. Neurosci.* 13 336–349. 10.1038/nrn3214 22498897

[B12] Catani M. Dell’Acqua F. Thiebaut D. S. M. (2013). A revised limbic system model for memory, emotion and behaviour. *Neurosci. Biobehav. Rev.* 37 1724–1737. 10.1016/j.neubiorev.2013.07.001 23850593

[B13] Chang S. W. C. Gariépy J. Platt M. L. (2012). Neuronal reference frames for social decisions in primate frontal cortex. *Nat. Neurosci.* 16 243–250. 10.1038/nn.3287 23263442 PMC3557617

[B14] Chen Y. Huang X. Wu M. Li K. Hu X. Jiang P. (2019). Disrupted brain functional networks in drug-nave children with attention deficit hyperactivity disorder assessed using graph theory analysis. *Hum. Brain Mapp.* 40 4877–4887. 10.1002/hbm.24743 31361385 PMC6865592

[B15] Christiansen H. Hirsch O. Albrecht B. Chavanon M. L. (2019). Attention-deficit/hyperactivity disorder (ADHD) and emotion regulation over the life span. *Curr. Psychiat. Rep.* 21:17. 10.1007/s11920-019-1003-6 30826879

[B16] Collins F. S. Varmus H. (2015). A new initiative on precision medicine. *New Engl. J. Med.* 372 793–795. 10.1056/NEJMp1500523 25635347 PMC5101938

[B17] Constantino J. N. (2013). *Social Responsiveness Scale: Encyclopedia of Autism Spectrum Disorders.* New York, NY: Springer.

[B18] Cui Z. Zhong S. Xu P. He Y. Gong G. (2013). PANDA: a pipeline toolbox for analyzing brain diffusion images. *Front. Hum. Neurosci.* 7:42. 10.3389/fnhum.2013.00042 23439846 PMC3578208

[B19] Di Martino A. Zuo X. N. Kelly C. Grzadzinski R. Mennes M. Schvarcz A. (2013). Shared and distinct intrinsic functional network centrality in autism and attention-deficit/hyperactivity disorder. *Biol. Psychiat.* 74 623–632. 10.1016/j.biopsych.2013.02.011 23541632 PMC4508007

[B20] Feis Y. F. (2010). “Wechsler intelligence scale for children-IV (WISC-IV),” in *Encyclopedia of Cross-Cultural School Psychology*, ed. Clauss-Ehlers C. S. (Boston, MA: Springer), 1030–1032. 10.1007/978-0-387-71799-9_446

[B21] Filippo M. D. Picconi B. Tantucci M. Ghiglieri V. Bagetta V. Sgobio C. (2009). Short-term and long-term plasticity at corticostriatal synapses: implications for learning and memory. *Behav. Brain Res.* 199 108–118. 10.1016/j.bbr.2008.09.025 18948145

[B22] Fonseca D. D. Seguier V. Santos A. Poinso F. Deruelle C. (2009). Emotion understanding in children with ADHD. *Child Psychiat. Hum. Dev.* 40 111–121. 10.1007/s10578-008-0114-9 18663570

[B23] Fox M. D. Snyder A. Z. Vincent J. L. Corbetta M. Van Essen D. C. Raichle M. E. (2005). From the Cover: the human brain is intrinsically organized into dynamic, anticorrelated functional networks. *Proc. Natl. Acad. Sci. U.S.A.* 102 9673–9678. 10.1073/pnas.0504136102 15976020 PMC1157105

[B24] Gau S. F. Shang C. Y. Liu S. K. Lin C. H. Swanson J. M. Liu Y.-C. (2008). Psychometric properties of the Chinese version of the Swanson, Nolan, and Pelham, version IV scale - parent form. *Int. J. Method Psychiatr. Res.* 17 35–44. 10.1002/mpr.237 18286459 PMC6878250

[B25] Gibbard C. R. Ren J. J. Skuse D. H. Clayden J. D. Clark C. A. (2017). Structural connectivity of the amygdala in young adults with autism spectrum disorder. *Hum. Brain Mapp.* 39 1270–1282. 10.1002/hbm.23915 29265723 PMC5838552

[B26] Gotts S. J. Kyle S. W. Milbury L. A. Wallace G. L. Cox R. W. Alex M. (2012). Fractionation of social brain circuits in autism spectrum disorders. *Brain* 135 2711–2725. 10.1093/brain/aws160 22791801 PMC3437021

[B27] Groen W. Teluij M. Buitelaar J. Tendolkar I. (2010). Amygdala and hippocampus enlargement during adolescence in autism. *J. Am. Acad. Child Psychiatry* 49 552–560. 10.1016/j.jaac.2009.12.023 20494265

[B28] Gudmundsson O. O. Walters G. B. Ingason A. Johansson S. Zayats T. Athanasiu L. (2019). Attention-deficit hyperactivity disorder shares copy number variant risk with schizophrenia and autism spectrum disorder. *Transl. Psychiatry* 9:258. 10.1038/s41398-019-0599-y 31624239 PMC6797719

[B29] Guo B. Chen J. Chen Q. Ren K. Feng D. Mao H. (2019). Anterior cingulate cortex dysfunction underlies social deficits in shank3 mutant mice. *Nat. Neurosci.* 22 1223–1234. 10.1038/s41593-019-0445-9 31332372

[B30] Guo X. N. Duan X. J. Long Z. L. Chen H. Wang Y. F. Zheng J. J. (2016). Decreased amygdala functional connectivity in adolescents with autism: a resting-state fMRI study. *Psychiat. Res. Neuroimage* 257 47–56. 10.1016/j.pscychresns.2016.10.005 27969061

[B31] Hagmann P. Cammoun L. Gigandet X. Meuli R. Honey C. J. Wedeen V. J. (2008). Mapping the structural core of Human Cereb. *Cortex PLoS Biol.* 6:e159. 10.1371/journal.pbio.0060159 18597554 PMC2443193

[B32] Hoogman M. Bralten J. Hibar D. P. Mennes M. Zwiers M. P. Schweren L. S. J. (2017). Subcortical brain volume differences in participants with attention deficit hyperactivity disorder in children and adults: a cross-sectional mega-analysis. *Lancet Psychiatry* 4 310–319. 10.1016/S2215-0366(17)30049-4 28219628 PMC5933934

[B33] Hoogman M. Muetzel R. Guimaraes J. P. Shumskaya E. Mennes M. Zwiers M. P. (2019). Brain imaging of the cortex in ADHD: a coordinated analysis of large-scale clinical and population-based samples. *Am. J. Psychiatry* 176 531–542. 10.1176/appi.ajp.2019.18091033 31014101 PMC6879185

[B34] Hulvershorn L. A. Mennes M. Castellanos F. X. Di Martino A. Milham M. P. Hummer T. A. (2014). Abnormal Amygdala functional connectivity associated with emotional lability in children with attention-deficit/hyperactivity disorder. *J. Am. Acad. Child Psychiatry* 53 351–361.e1. 10.1016/j.jaac.2013.11.012 24565362 PMC3961844

[B35] Iidaka T. Kogata T. Mano Y. Komeda H. (2019). Thalamocortical hyperconnectivity and amygdala-cortical hypoconnectivity in male patients with autism spectrum disorder. *Front. Psychiatry* 10:252. 10.3389/fpsyt.2019.00252 31057443 PMC6482335

[B36] Iturria-Medina Y. Sotero R. C. Canales-Rodríguez E. J. Alema’n-Go’mez Y. Melie-García L. (2008). Studying the human brain anatomical network via diffusion-weighted MRI and graph theory. *Neuroimage* 40 1064–1076. 10.1016/j.neuroimage.2007.10.060 18272400

[B37] Just M. A. Cherkassky V. L. Keller T. A. Minshew N. J. (2004). Cortical activation and synchronization during sentence comprehension in high-functioning autism: evidence of underconnectivity. *Brain* 127 (Pt 8) 1811–1821. 10.1093/brain/awh199 15215213

[B38] Kernbach J. M. Satterthwaite T. D. Bassett D. S. Jonathan S. Daniel M. Sarah K. (2018). Shared endo-phenotypes of default mode dysfunction in attention deficit/hyperactivity disorder and autism spectrum disorder. *Transl. Psychiatry* 8:133. 10.1038/s41398-018-0179-6 30018328 PMC6050263

[B39] Konrad K. Eickhoff S. B. (2010). Is the ADHD brain wired differently? A review on structural and functional connectivity in attention deficit hyperactivity disorder. *Hum. Brain Mapp.* 31 904–916. 10.1002/hbm.21058 20496381 PMC6871159

[B40] Lee J. M. Kyeong S. Kim E. Cheon K. (2016). Abnormalities of Inter- and Intra-hemispheric functional connectivity in autism spectrum disorders: a study using the autism brain imaging data exchange database. *Front. Neurosci.* 10:191. 10.3389/fnins.2016.00191 27199653 PMC4853413

[B41] Li S. J. Wang Y. Qian L. Liu G. Liu S. F. Zou L. P. (2018). Alterations of white matter connectivity in preschool children with autism spectrum disorder. *Radiology* 288:170059. 10.1148/radiol.2018170059 29584599

[B42] Lord C. Rutter M. Couteur L. A. (1994). Autism diagnostic interview-revised: a revised version of a diagnostic interview for caregivers of individuals with possible pervasive developmental disorders. *J. Autism Dev. Disord.* 24 659–685. 10.1007/BF02172145 7814313

[B43] Lord C. Rutter M. Goode S. Heemsbergen J. Jordan H. Mawhood L. (1989). Autism diagnostic observation schedule: a standardized observation of communicative and social behavior. *J. Autism Dev. Disord.* 19 185–212. 10.1007/bf02211841 2745388

[B44] Medin T. Rinholm J. E. Owe S. G. Sagvolden T. Gjedde A. Storm-Mathisen J. (2013). Low dopamine D5 receptor density in hippocampus in an animal model of attention-deficit/hyperactivity disorder (ADHD). *Neuroscience* 242 11–20. 10.1016/j.neuroscience.2013.03.036 23541742

[B45] Mesulam M. M. (1998). From sensation to cognition. *Brain* 121 (Pt 6) 1013–1052. 10.1093/brain/121.6.1013 9648540

[B46] Miller M. Hanford R. B. Fassbender C. Duke M. Schweitzer J. B. (2011). Affect recognition in adults with ADHD. *J. Attention Disord.* 15 452–460. 10.1177/1087054710368636 20555036 PMC3950374

[B47] Monk C. S. Peltier S. J. Wiggins J. L. Weng S. J. Carrasco M. Risi S. (2009). Abnormalities of intrinsic functional connectivity in autism spectrum disorders. *Neuroimage* 47 764–772. 10.1016/j.neuroimage.2009.04.069 19409498 PMC2731579

[B48] Mori S. van Zijl P. C. M. (2002). Fiber tracking: principles and strategies - a technical review. *NMR Biomed.* 15 468–480. 10.1002/nbm.781 12489096

[B49] Mundy P. (2017). A review of joint attention and social-cognitive brain systems in typical development and autism spectrum disorder. *Eur. J. Neurosci.* 47 497–514. 10.1111/ejn.13720 28922520

[B50] Odriozola P. Dajani D. R. Burrows C. A. Gabard-Durnam L. J. Goodman E. Baez A. C. (2018). Atypical frontoamygdala functional connectivity in youth with autism. *Dev. Cogn. Neurosci.* 37:100603. 10.1016/j.dcn.2018.12.001 30581125 PMC6570504

[B51] Pagani M. Manouilenko I. Stone-Elander S. Odh R. Salmaso D. Hatherly R. (2012). Brief report: alterations in cerebral blood flow as assessed by PET/CT in adults with autism spectrum disorder with normal IQ. *J. Autism Dev. Disord.* 42 313–318. 10.1007/s10803-011-1240-y 21487836

[B52] Paquette V. Lévesque J. Mensour B. Leroux J. M. Beaudoin G. Bourgouin P. (2003). “Change the mind and you change the brain”: effects of cognitive-behavioral therapy on the neural correlates of spider phobia. *Neuroimage* 18 401–409. 10.1016/s1053-8119(02)00030-7 12595193

[B53] Posner J. Nagel B. J. Maia T. V. Mechling A. Oh M. Wang Z. (2011). Abnormal amygdalar activation and connectivity in adolescents with attention-deficit/hyperactivity disorder. *J. Am. Acad. Child Psychiatry* 50 828–837.e3. 10.1016/j.jaac.2011.05.010 21784302 PMC3155780

[B54] Posner J. Siciliano F. Wang Z. Liu J. Sonuga-Barke E. Greenhill L. (2014). A multimodal MRI study of the hippocampus in medication-naive children with ADHD: what connects ADHD and depression? *Psychiat. Res. Neuroimage* 224 112–118. 10.1016/j.pscychresns.2014.08.006 25220159 PMC4195849

[B55] Qian L. Wang Y. Chu K. Li Y. Xiao C. Xiao T. (2018). Alterations in hub organization in the white matter structural network in toddlers with autism spectrum disorder: a 2-year follow-up study. *Autism Res.* 11 1218–1228. 10.1002/aur.1983 30114344

[B56] Roine U. Roine T. Salmi J. Taina N. W. Tani P. Leppämäki S. (2015). Abnormal wiring of the connectome in adults with high-functioning autism spectrum disorder. *Mol. Autism.* 6:65. 10.1186/s13229-015-0058-4 26677408 PMC4681075

[B57] Rommelse N. N. J. Franke B. Geurts H. M. Hartman C. A. Buitelaar J. K. (2010). Shared heritability of attention-deficit/hyperactivity disorder and autism spectrum disorder. *Eur. Child Adoles. Psychiatry* 19 281–295. 10.1007/s00787-010-0092-x 20148275 PMC2839489

[B58] Rommelse N. N. J. Geurts H. M. Franke B. Buitelaar J. K. Hartman C. A. (2011). A review on cognitive and brain endophenotypes that may be common in autism spectrum disorder and attention-deficit/hyperactivity disorder and facilitate the search for pleiotropic genes. *Neurosci. Biobehav. Rev.* 35 1363–1396. 10.1016/j.neubiorev.2011.02.015 21382410

[B59] Rubinov M. Sporns O. (2010). Complex network measures of brain connectivity: uses and interpretations. *Neuroimage* 52 1059–1069. 10.1016/j.neuroimage.2009.10.003 19819337

[B60] Rudie J. D. Brown J. A. Beck-Pancer D. Hernandez L. M. Dennis E. L. Thompson P. M. (2013). Altered functional and structural brain network organization in autism. *Neuroimage Clin.* 2 79–94. 10.1016/j.nicl.2012.11.006 24179761 PMC3777708

[B61] Schoch H. Kreibich A. S. Ferri S. L. White R. S. Bohorquez D. Banerjee A. (2016). Sociability deficits and altered amygdala circuits in mice lacking Pcdh10. *Ann. Autism Assoc. Gene Biol. Psychiatry* 81 193–202. 10.1016/j.biopsych.2016.06.008 27567313 PMC5161717

[B62] Schumann C. M. Hamstra J. Goodlin-Jones B. L. Lotspeich L. J. Amaral D. G. (2004). The amygdala is enlarged in children but not adolescents with autism; the hippocampus is enlarged at all ages. *J. Neurosci.* 24:6392. 10.1523/JNEUROSCI.1297-04.2004 15254095 PMC6729537

[B63] Shu N. Liu Y. Li K. Duan Y. Wang J. Yu C. (2011). Diffusion tensor tractography reveals disrupted topological efficiency in white matter structural networks in multiple sclerosis. *Cereb. Cortex* 21 2565–2577. 10.1093/cercor/bhr039 21467209

[B64] Sidlauskaite J. Caeyenberghs K. Sonuga-Barke E. Roeyers H. Wiersema J. R. (2015). Whole-brain structural topology in adult attention-deficit/hyperactivity disorder: preserved global - disturbed local network organization. *Neuroimage Clin.* 9 506–512. 10.1016/j.nicl.2015.10.001 26640763 PMC4630025

[B65] Sporns O. Tononi G. Kotter R. (2005). The human connectome: a structural description of the human brain. *PLoS Comput. Biol.* 1:e42. 10.1371/journal.pcbi.0010042 16201007 PMC1239902

[B66] Stergiakouli E. Smith G. D. Martin J. Skuse D. H. Viechtbauer W. Ring S. M. (2017). Shared genetic influences between dimensional ASD and ADHD symptoms during child and adolescent development. *Mol. Autism.* 8:18. 10.1186/s13229-017-0131-2 28392908 PMC5379648

[B67] Sterley T. L. Howells F. M. Russell V. A. (2013). Evidence for reduced tonic levels of GABA in the hippocampus of an animal model of ADHD, the spontaneously hypertensive rat. *Brain Res.* 1541 52–60. 10.1016/j.brainres.2013.10.023 24161405

[B68] Supekar K. Musen M. Menon V. (2009). Development of large-scale functional brain networks in children. *PLoS Biol.* 7:e1000157. 10.1371/journal.pbio.1000157 19621066 PMC2705656

[B69] Takashi I. Takashi Y. Hiromi W. Motoaki N. Daiki J. Seiji S. (2014). Altered network topologies and hub organization in adults with autism: a resting-state fMRI study. *PLoS One* 9:e94115. 10.1371/journal.pone.0094115 24714805 PMC3979738

[B70] Travers B. G. Adluru N. Ennis C. Tromp do P. M. Destiche D. Doran S. (2012). Diffusion tensor imaging in autism spectrum disorder: a review. *Autism Res.* 5 289–313. 10.1002/aur.1243 22786754 PMC3474893

[B71] Tzourio-Mazoyer N. Landeau B. Papathanassiou D. Crivello F. Etard O. Delcroix N. (2002). Automated anatomical labeling of activations in SPM using a macroscopic anatomical parcellation of the MNI MRI single-subject brain. *Neuroimage* 15 273–289. 10.1006/nimg.2001.0978 11771995

[B72] Van der Meer J. M. J. Oerlemans A. M. van Steijn D. J. Lappenschaar M. G. A. de Sonneville L. M. J. Buitelaar J. K. (2012). Are autism spectrum disorder and attention-deficit/hyperactivity disorder different manifestations of one overarching disorder? cognitive and symptom evidence from a clinical and population-based sample. *J. Am. Acad. Child Psychiatry* 51 1160–1172.e3. 10.1016/j.jaac.2012.08.024 23101742

[B73] Van Rooij D. Anagnostou E. Arango C. Auzias G. Behrmann M. Busatto G. F. (2018). Cortical and subcortical brain morphometry differences between patients with autism spectrum disorder and healthy individuals across the lifespan: results from the ENIGMA ASD working group. *Am. J. Psychiatry* 175 359–369. 10.1176/appi.ajp.2017.17010100 29145754 PMC6546164

[B74] Vijayakumar N. Whittle S. Dennison M. Yücel M. Simmons J. Allen N. B. (2014). Development of temperamental effortful control mediates the relationship between maturation of the prefrontal cortex and psychopathology during adolescence: a 4-year longitudinal study. *Dev. Cogn. Neurosci.* 9 30–43. 10.1016/j.dcn.2013.12.002 24486655 PMC6989743

[B75] Wang L. Zhu C. He Y. Zang Y. Zang Y. Cao Q. (2009). Altered small-world brain functional networks in children with attention deficit/hyperactivity disorder. *Hum. Brain Mapp.* 30 638–649. 10.1002/hbm.20530 18219621 PMC6870909

[B76] Weng S. J. Wiggins J. L. Peltier S. J. Carrasco M. Risi S. Lord C. (2010). Alterations of resting state functional connectivity in the default network in adolescents with autism spectrum disorders. *Brain Res.* 1313 202–214. 10.1016/j.brainres.2009.11.057 20004180 PMC2818723

[B77] Xia S. Foxe J. J. Sroubek A. E. Branch C. Li X. (2014). Topological organization of the “small-world” visual attention network in children with attention deficit/hyperactivity disorder (ADHD). *Front. Hum. Neurosci.* 8:162. 10.3389/fnhum.2014.00162 24688465 PMC3960496

[B78] Zalesky A. Fornito A. Bullmore E. T. (2010). Network-based statistic: identifying differences in brain networks. *Neuroimage* 53 1197–1207. 10.1016/j.neuroimage.2010.06.041 20600983

[B79] Zielinski B. A. Anderson J. S. Froehlich A. L. Prigge M. B. D. Nielsen J. A. Cooperrider J. R. (2012). scMRI reveals large-scale brain network abnormalities in autism. *PLoS One* 7:e49172. 10.1371/journal.pone.0049172 23185305 PMC3504046

